# The Effect of Foot Reflexology Massage on Delirium and Physiological Indicators in Children Admitted to Pediatric Intensive Care Unit: A Randomized Clinical Trial

**DOI:** 10.1155/tswj/2352440

**Published:** 2026-04-26

**Authors:** Zahra Mazhari, Ali Manafi Anari, Farshad Heidari-Beni, Mona Alinejad-Naeini

**Affiliations:** ^1^ Pediatric and Intensive Neonatal Nursing Department, School of Nursing and Midwifery, Iran University of Medical Sciences, Tehran, Iran, iums.ac.ir; ^2^ Department of Pediatrics, Ali Asghar Children Hospital, Iran University of Medical Sciences, Tehran, Iran, iums.ac.ir; ^3^ Community-Oriented Nursing Midwifery Research Center, Nursing and Midwifery School, Shahrekord University of Medical Sciences, Shahrekord, Iran, skums.ac.ir

**Keywords:** delirium, foot reflexology massage, pediatric intensive care unit, physiological indicators

## Abstract

**Introduction:**

Children in pediatric intensive care units (PICUs) are commonly affected by delirium, which poses considerable challenges in clinical management. Changes in vital signs can serve as early warning indicators of disease progression in children. Nonpharmacological interventions in the handling of delirium and improvement of physiological indicators include various types of massage. This study was aimed at investigating the effect of foot reflexology massage on delirium and physiological indicators in children admitted to the PICU.

**Methods:**

This randomized clinical trial was directed in the PICU of Ali Asghar Hospital, affiliated with Iran University of Medical Sciences, Tehran, Iran. The study randomly assigned a total of 74 participants to either an intervention group or a control group. Foot reflexology massage was administered to the intervention group. Information was captured before and after the intervention using tools assessing demographic information/data, physiological indicators, and pediatric delirium (Cornell tool).

**Results:**

The analysis revealed that on the second day, the frequency of delirium in the intervention group was significantly lower than that in the control group after the intervention (*p* = 0.004). Also, immediately after the intervention, heart rate, diastolic blood pressure, and systolic blood pressure in the intervention group were significantly lower than those in the control group (*p* < 0.001, *p* = 0.026, and *p* = 0.002, respectively). Also, the results showed that there was no statistically significant difference in arterial blood oxygen saturation between the two groups after the intervention (*p* = 0.345).

**Conclusion:**

The evidences of our study show that foot reflexology can decrease delirium and regulate heart rate and blood pressure, but its effect on arterial blood oxygen saturation requires further investigation.

## 1. Introduction

Every year, more than 300,000 children suffer injuries or life‐threatening illnesses, are admitted to the PICU, and require the highest level of care [1]. Hospitalization of children in the PICU is associated with complications such as delirium [2]. Prevalence rates of pediatric delirium in the PICU range from 10% to 30%, and after cardiac surgery, it can reach 40% [3]. In the study by Navaeifar et al., the prevalence rate of pediatric delirium in Iran was 25% [4].

Without proper diagnosis and treatment, delirium has been linked to negative consequences including more illness, longer medical center stays, increased healthcare expenditures and persistent cognitive deficits, prolonged mechanical ventilation, and increased mortality [3, 5]. Delirium also causes anxiety in the patient, family, and healthcare providers [6]. The study of delirium in children has not received as much attention from therapists and researchers as it deserves. Therefore, greater attention to the diagnosis and treatment of delirium is necessary [7]. Delirium treatment is based on pharmacological and nonpharmacological interventions. Drug‐based treatments often lead to a higher rate of occurrence of restlessness [8]. Nonpharmacological management is necessary to reduce delirium [9] including family participation, environmental interventions such as reducing light and noise during sleep, setting monitor alarm volume levels, limit the use of medical equipment and keep conversations to a minimum near the child′s room, establishing a daily routine for sleep and wakefulness, reducing the use of restraints, encouraging early movement, play, and various types of massage [10–12]. There is a considerable evidence regarding the effect of therapeutic modalities without medication in the prevention and treatment of delirium; however, the effectiveness of various kinds of interventions, including therapeutic touch and massage, has not been extensively investigated in the literature [13].

Variations in vital signs serve a dual purpose: They reflect current physiological functioning and offer an early alert mechanism for worsening clinical conditions [14]. In conjunction with pathophysiological mechanisms, cognitive and emotional factors also have an effect on changes in vital parameters. Psychological disorders include chronic stress, anxiety, depression, and anger [15] Delirium, in turn, is associated with changes in physiological parameters, cognitive changes, and vulnerability [16]. There is also a method of detecting delirium based on vital signs, as discussed in the study by Kuhn et al. [17]. Treatment staff use both pharmacological interventions alongside nonpharmacological strategies for management to control anxiety and improve patients′ physiological indicators [18].

Today, considerable emphasis is placed on the use of complementary medicine in the healthcare system [19]. Over the past decade, nonpharmacological methods have attracted the attention and interest of patients and their families because they are low cost, effective, and efficient and demonstrate favorable safety characteristics, including diminished adverse effects, noninvasiveness, and a nonaddictive nature [20]. One of these nonpharmacological methods, which involves tactile sensory stimulation, is foot reflexology massage [21]. Reflexology massage includes pressure and massage on specific points. Based on Dr. FitzGerald′s theoretical framework, reflex massage posits that a vital force or life energy circulates through specific pathways from the feet to the internal organs, and the purpose of this massage is to eliminate congestion and release the flow of energy, thereby improving diseases. Additionally, by affecting the nervous and muscular systems, it increases blood flow, enhances the excretion of waste materials, and improves blood flow to the organs and brain [22]. Based on the theory of hemodynamics, reflex massage stimulation enhances circulation both locally, in the massaged region, and systemically throughout the body, helping to increase relaxation and improve treatment [23]. Various studies have introduced foot reflexology massage as a noninvasive nursing intervention in children [24–26].

According to the above explanations and considering that delirium frequently occurs in children who are hospitalized in the and is associated with long‐term psychological disorders as well as mortality, it seems useful to conduct this study. There is very little information about delirium in children, mainly because it is often not considered in this population [4, 7].

Among the foremost priorities in the PICU is to improve the patient care quality to achieve the best outcomes and enhance recovery in critically ill children. By improving nursing care for sick children, it is possible to reduce mortality rates and related complications [27]. More interventional scholarships are also required to appraise the effectiveness of interventions aimed at minimizing delirium in pediatric patients and to formulate international clinical guidelines for its prevention and management in critically ill children [12]. Therefore, the primary objective of this study is to assess the impact of foot reflexology massage on delirium and physiological parameters in children admitted to the PICU.

## 2. Materials and Methods

### 2.1. Design and Population

**Figure 1:**
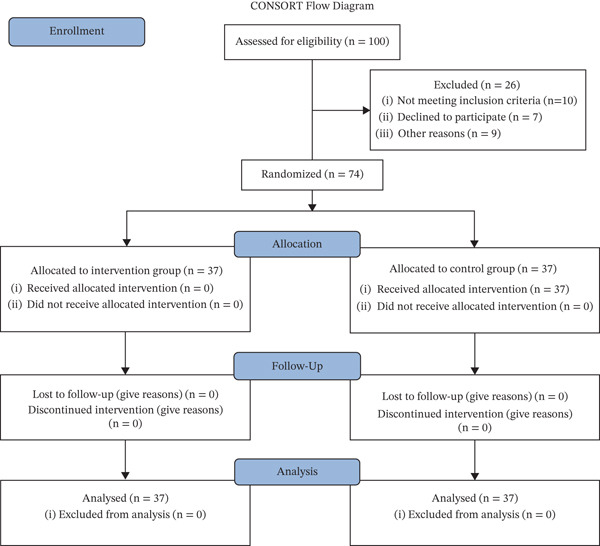
The flowchart of sample selection.

This randomized controlled clinical trial was conducted in the PICU of Iran University of Medical Sciences between March and August 2024. Participants were included according to these criteria: children aged 6–12 years, failure to score −4 or −5 on the Richmond Agitation Scale, hospitalization in the PICU for at least 3 days, normal skin integrity without hyperkeratotic conditions like corns, wounds, previous scars, burns, amputations, and skin diseases on the legs, absence of vessel trauma in the lower extremities with no sensory or motor deficits in the legs (confirmed by a neurologist), diagnosis of delirium by obtaining a delirium score of 9 or above based on the Cornell tool and a pediatric neurologist, no previous exposure to foot reflexology interventions, and confirmation of the diagnosis by a pediatric specialist. Children were excluded if they had any of the following: (i) mechanical ventilation, (ii) restlessness, (iii) lack of proper cooperation or resistance from the child or parents at any time during the study, (iv) under critical circumstances, notably respiratory/cardiac arrest or convulsive episodes, (v) acute heart or respiratory diseases, or (vi) reduction of vital signs beyond expected limits, such as a drop in blood pressure (below the 25th percentile) and bradycardia (below 65 beats per minute). A power analysis (95% CI, 80% power) was performed, estimating an effect size of 0.5 for the influence of foot reflexology on pediatric delirium, to derive the necessary sample size. The sample was calculated as 32 children per group. To account for potential sample drop, 15% was added. Therefore, the final cohort size for each group was established as 37 pediatric participants (Figure 1).

### 2.2. Outcome Measures

A three‐part instrument was employed for data collection, comprising (1) a demographic questionnaire for pediatric participants, (2) the Cornell Assessment of Pediatric Delirium (CAPD), and (3) a clinical observation form for recording physiological indicators. The demographic data form (demographic information) comprised age, sex, children′s body weight, initial diagnosis of the child′s illness, child′s level of education, history of hospitalization, history of hospitalization in the PICU, duration of illness, history of drug use, and underlying diseases.

In pediatric intensive care, the CAPD is widely utilized as a diagnostic measure for delirium [28, 29]. This scale was designed as a brief, nurse‐friendly screening instrument to promptly identify pediatric patients across all age that vulnerable to delirium in intensive care units [30, 31]. The scale comprises eight items aligned with the DSM‐5 diagnostic criterion for disturbances in awareness and cognition, while also incorporating psychomotor manifestations. Extensive testing has shown it is highly feasible for bedside nurses to administer and can be done in under 2 min for each patient. Since the assessment is grounded in behavioral observations made during standard nursing care, it does not necessitate patient engagement at the time of observation, leading to its widespread tolerability [31]. Using a 0–4 scoring system across eight observable items, the CAPD identifies delirium with a threshold score of ≥ 9. Assessments of children below 24 months should be performed with prudence and in combination with developmental screening, the authors provided an auxiliary table of developmental anchor points, organized into seven age‐based columns: newborn, 4, 6, 8, and 28 weeks, and 1 and 2 years. Psychometric evaluation revealed that the CAPD achieved a sensitivity of 94.1% (95% CI: 83.8%–98.8%) and specificity of 79.2% (95% CI: 73.5%–84.9%). The measure also established robust internal consistency, with a total Cronbach′s *α* of 0.90 and item‐level consistency between 0.87 and 0.90 [31]. The psychometric properties of this instrument were formally assessed in the Iranian context by Navaeifar et al. in 2012. The degree of agreement among raters was examined using Kendall′s test. The resulting Kendall′s coefficient for the total score was 0.046, demonstrating no statistically significant variation across raters for each of the eight questions or the final Cornell tool score [4].

The observation form for physiological indicators was compiled by the researcher and included heart rate (beats per minute), blood pressure (mmHg), and arterial oxygen saturation level, measured via the monitor connected to the child. Measurements were recorded in chronological order.

To evaluate interrater reliability, Cohen′s kappa was computed via SPSS (v. 23.0). The kappa statistic measures agreement between observers on a scale from 0 to 1, with a minimum threshold of > 0.6 for acceptable reliability and > 0.8 reflecting excellent concordance [32]. In this study, 10 independent observations were conducted by two rates, and interrater reliability was assessed using Cohen′s kappa, yielding a coefficient of 0.97—indicating ideal agreement.

### 2.3. Randomization and Allocation

After determining the eligibility of the children who were admitted to the PICU, allocation was performed. We accidentally allocated participants to the intervention or control group. We utilized a computer‐based random allocation sequence (https://www.randomizer.org/) with random permuted block sizes of 4 or 6. The massage researcher and the pediatrician were kept blinded to the allocation. Information regarding allocation was available only to one researcher.

### 2.4. Intervention

Ethical permission was permitted by the university ethics committee before our study, the researcher entered the research environment. He then clearly explained the research procedures and objectives to the supervisor and the doctor in charge of the department. Sampling was continuous and based on the inclusion criteria. Initially, samples meeting the inclusion criteria were selected, and the child′s agitation score was measured using the Richmond Agitation Scale. If the child achieved a score between −3 and +4, delirium cruelty was measured to calculate the delirium score. Children exhibiting a delirium score of 9 or higher were identified, and, with the doctor′s confirmation of a delirium diagnosis, the selected sample was included in the study. Then, using computer randomization (https://www.randomizer.org/), the selected children were assigned to the control and intervention groups (foot reflex massage intervention). The objectives of study and the foot reflexology massage were explained orally, and an educational video was shown to the parents. All their questions about the massage were answered. Consent was acquired in two forms: verbal assent from the child and written informed consent from the parents. The demographic data were then recorded by the investigator from the child′s medical record.

In cases where the child was assigned to the intervention group, as well as receiving usual care, foot reflexology massage was also made. Before the foot reflexology massage, the researcher first ready the child and the setting for the intervention. The child was first connected to cardiorespiratory monitoring, and then, 5 min before the foot reflexology massage, the physiological indicators form (blood pressure, heart rate, and arterial blood oxygen saturation) and the Cornell Delirium Questionnaire for Children (CAPD) were completed. The massage lasted for 28 min. After the massage, the CAPD was completed again 2 h later. For children in the control group, the CAPD was completed 2 h and 28 min after the initial assessment. The intervention and delirium measurement continued for 2 days. Immediately after the reflexology massage, the physiological indicators form was completed again, and the CAPD was completed 2 h later.

It should be noted that in this study, the massage was done by a researcher who had established 3 months of certification in foot reflexology massage under the supervision of a specialist in physical medicine, rheumatology, and rehabilitation. She correctly identified the reflex points of the foot, learned methods for applying force, and obtained certification.

### 2.5. Massage Therapy

In this research, foot reflexology massage is performed in the following steps.

First, the child and their mother were prepared psychologically by the researcher, who explained the purpose, effects, and importance of foot reflexology.

i. Preparation stage: The children were placed lying on their backs with the head of the bed raised. Children′s feet were positioned comfortably, a compact cushion was placed under the knees to make the massage easier to perform. The child′s feet were then washed with shampoo and dried with a towel. The researcher′s hands were warmed before starting the session. The researcher was seated in front of the children′s feet. Three drops of baby oil were applied to the researcher′s hands to facilitate the massage and minimize skin friction on the children′s feet.
ii. Warm‐up and relaxation steps: Reflexology began with the relaxation technique involving rubbing the feet by lightly running the fingertips up, down, and along the sides of each foot in a feather‐like motion.
iii. Use circular motions to massage the reflex areas on the soles: Foot reflexology was applied in a top‐to‐down manner, with the actual massage starting at the head/brain (top of each toe) pituitary (center of the big toe), thyroid (base of the big toe), neck/shoulders (ridge of the toes), chest/lungs (ball of the foot), and kidney (base of the toe to base of heel) to stabilize physiological parameters and relieve fatigue. Each point was pressed and released; apply the thumb walking method for approximately 2–3 min. Force was then exerted to the solar plexus reflex point by placing the thumb in the center of the foot to relieve stress. After reflexology techniques were applied to selected areas corresponding to the nerve supply on the feet [33].

### 2.6. Blinding/Masking

Blinding of clinicians was not possible in this study by reason of the massage intervention. However, masking was implemented during data analysis. To ensure blinding in outcome assessment, the data were coded in the software, and analysis was conducted by a statistician who was unaware of the coding and did not know which data corresponded to the intervention or control conditions.

### 2.7. Statistical Analysis

Data were analyzed utilizing SPSS (Statistical Package for the Social Sciences) Software Version 23 IBM Corp., Chicago, Illinois. Descriptive statistics as well as mean, standard deviation, frequency, and percentage were accomplished for all parameters. Inferential statistics including independent *t*‐test, chi‐square test, Fisher′s exact test, analysis of covariance (ANCOVA), and Mann–Whitney *U* test were performed to explore differences between groups. Results were measured statistically significant at *p* < 0.05.

## 3. Results

Most children in both groups were male, had a history of drug use, no underlying disease, a disease duration was less than 1 year, a history of hospitalization, a hospital stay of less than 3 days, and no history of admission to the pediatric ICU. Additionally, most children in the control group were under 8 years old, had preschool education, and weighed less than 30 kg, while most children in the intervention group were over 10 years old, weighed more than 30 kg, and had a sixth‐grade education level. However, no statistically significant differences were detected in the demographic characteristics between the two groups. The demographic characteristics of the children are offered in Table 1.

**Table 1:** Demographic characteristics of children.

Variable	Groups				
	Control		Intervention		*p* value
	*N*	%	*N*	%	
Gender					0.483^€^
Female	18	48.6	15	40.5	
Male	19	51.4	22	59.5	
Age (year)					*p* = 0.179^ⴄ^
Under 8	16	43.2	12	32.4	
8–10	10	27	9	24.3	
Over 8	11	29.7	16	43.2	
Weight (kg)					*p* = 0.670^ⴄ^
Under 30	20	54.1	16	43.2	
30 and more	17	45.9	21	56.8	
Educational level					*p* = 0.765^∗∗^
Preschool	13	35.1	8	21.6	
First grade	3	8.1	3	8.1	
Second grade	4	10.8	6	16.2	
Third grade	3	8.1	3	8.1	
Fourth grade	3	8.1	2	5.4	
Fifth grade	5	13.5	4	10.8	
Sixth grade	6	16.2	11	29.7	
History of medication use					*p* = 0.116^€^
Yes	15	40.5	9	24.3	
No	22	59.5	28	75.7	
Underlying disease					*p* = 0.136^€^
Yes	15	40.5	9	24.3	
No	22	59.5	28	75.7	
Duration of disease (year)					*p* = 0.109^∗^
Under 1	29	78.4	32	86.5	
1–5	6	16.2	4	10.8	
6 and more	2	5.4	1	2.7	
History of hospitalization					*p* = 0.197^€^
Yes	29	78.4	24	64.9	
No	8	21.6	13	35.1	
History of hospitalization at NICU					*p* = 0.619^€^
Yes	13	35.1	11	29.7	
No	24	64.9	26	70.3	
Disease type					*p* = 0.084^∗∗^
Infective	8	21.6	7	18.9	
Respiratory	6	16.2	4	10.8	
Digestive	4	10.8	0	0	
Neurologic	6	2/16	3	8.1	
Other	13	35.1	23	62.2	
Duration of hospitalization(day)					*p* = 0.159^∗^
3–6	25	67.7	31	83.8	
6 and more	12	32.4	6	16.2	

^∗^Mann–Whitney.  ^∗∗^Fisher exact test. ^€^Chi‐square. ^ⴄ^Independent *t*‐test.

As shown in Tables 2 and 3 on Day 1, before the intervention, all children in the control and intervention groups had delirium. Immediately after the intervention, 94.6% of children in the control group and 86.5% in the intervention group had delirium. The results of the chi‐square test indicated that this difference was not statistically significant (*p* = 0.430). On the second day before the intervention, 97.3% of children in the control group and 91.9% in the intervention group had delirium, while immediately after the intervention, 94.6% in the control group and 75.7% in the intervention group had delirium. The results presented a significant difference in the frequency of delirium between groups at this time (*p* = 0.022). The same trend was observed in the severity of delirium. While there was no significant difference between the two groups in terms of delirium severity on the first day before and after the intervention (*p* = 0.276 *and* *p* = 0.122) and on the second day before the intervention (*p* = 0.445), a significant difference was observed between the two groups on the second day after the intervention (*p* < 0.001). The effect sizes between groups after the intervention on first day was small (ⴄ2 = 0.021) and in the second day was large (ⴄ2 = 0.190).

**Table 2:** The frequency of delirium on the first and second days in the control and intervention groups.

Variable	Time			
	First day		Second day	
	Before the intervention (%) *N*	After the intervention (%) *N*	Before the intervention (%) *N*	After the intervention (%) *N*
Delirium				
Control	37 (100)	35 (94.6)	36 (97.3)	35 (94.6)
Intervention	37 (100)	32 (86.5)	34 (91.9)	28 (75.7)
*p* value	−	0.430	0.615	0.022

**Table 3:** The severity of delirium on the first and second days in the control and intervention groups.

Variable	Time			
	First day		Second day	
	Before the intervention (*m* *e* *a* *n* ± *S* *D*)	After the intervention (*m* *e* *a* *n* ± *S* *D*)	Before the intervention (*m* *e* *a* *n* ± *S* *D*)	After the intervention (*m* *e* *a* *n* ± *S* *D*)
Delirium				
Control	13.73 ± 3.98	13.84 ± 4.88	14.62 ± 4.25	14.16 ± 5.02
Intervention	12.70 ± 4.07	12.03 ± 4.69	13.84 ± 4.52	10.57 ± 4.34
*p* value	0.276	0.122	0.445	< 0.001
Partial eta squared (ⴄ^2^)	—	0.021	—	0.190

Also, the trend of delirium severity across different times within and between groups was analyzed using three‐way repeated‐measure ANOVAs with groups as between‐subject factor and times (before and after the interventions) and days (first and second days) as within‐subject factors. The result showed that within the subject analysis, interaction of day and group was not significant (*p* = 0464) but interaction of time and group and interaction of day and time and group was significant (*p* = 0.001 and 0.032, respectively). Also, in between‐subject analysis, the group effect was significant (*p* = 0.033) (Figure 2).

Figure 2Trends in delirium severity on the first and second days in the control and intervention groups. (a) First day. (b) Second day.

**Figure:**
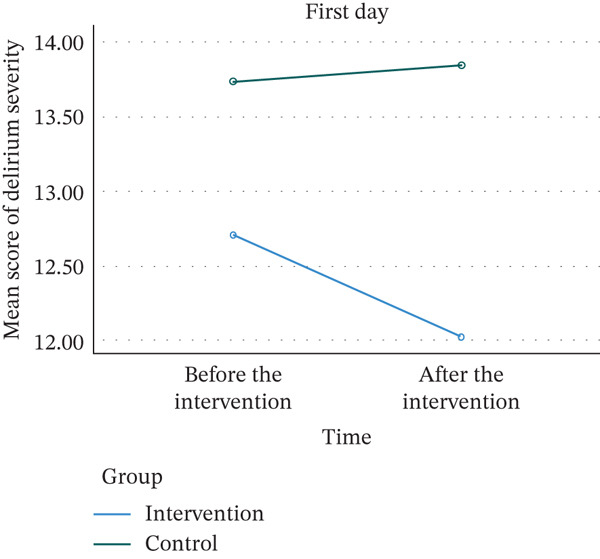
(a)

**Figure:**
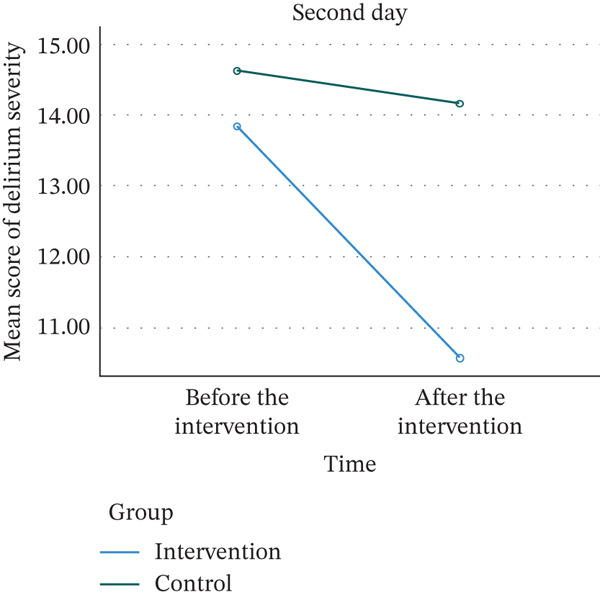
(b)

As illustrated in Table 4, the results of the independent *t*‐test indicated that Day 1, before the intervention, there was no statistically significant difference in heart rate between the two groups (*p* = 0.899). The ANCOVA showed that immediately after the intervention, the mean heart rate in the intervention group was significantly lower than that in the control group (*p* < 0.001). The same trend was observed on the second day; there was no statistically significant difference in mean heart rate between the two groups before the intervention (*p* = 0.497), but immediately after the intervention, the mean heart rate in the intervention group was significantly lower than that in the control group (*p* < 0.001). The effect sizes between groups after the intervention on both first and second days were large (ⴄ2 = 0.200 and 0.183, respectively).

**Table 4:** The trend of delirium severity across different times, within and between groups.

Variable	*D* *a* *y*∗*g* *r* *o* *u* *p*	*T* *i* *m* *e*∗*g* *r* *o* *u* *p*	*D* *a* *y*∗*t* *i* *m* *e*∗*g* *r* *o* *u* *p*	Group
Within‐subject effect (*p* value)	0.464	0.001	0.032	
Between‐subject effect (*p* value)				0.033

As outlined in Table 5, the results of the independent *t*‐test showed that there was no statistically significant difference in mean diastolic blood pressure between the two groups on the first day before the intervention (*p* = 0.517). However, the ANCOVA showed that immediately after the start of the intervention, the mean diastolic blood pressure was significantly lower in the intervention group than in the control group (*p* = 0.011). The same trend of the first day was observed on the second day: The mean diastolic blood pressure between the two groups before the intervention was not statistically significant (*p* = 0.757), but instantly after the intervention, the mean diastolic blood pressure in the intervention group was significantly lower than that in the control group (*p* = 0.026). The effect sizes between groups after the intervention on both first and second days were medium (ⴄ2 = 0.087 and 0.068, respectively).

**Table 5:** The mean heart rate, diastolic blood pressure, systolic blood pressure, and oxygen saturation on the first and second days in the control and intervention groups.

Variable	Time			
	First day		Second day	
	Before the intervention (*m* *e* *a* *n* ± *S* *D*)	After the intervention (*m* *e* *a* *n* ± *S* *D*)	Before the intervention (*m* *e* *a* *n* ± *S* *D*)	After the intervention (*m* *e* *a* *n* ± *S* *D*)
Heart rate				
Control	106.54 ± 16.73	105.35 ± 16.81	107.95 ± 17.63	105.00 ± 17.82
Intervention	106.08 ± 14.09	95.86 ± 14.58	105.32 ± 15.27	94.16 ± 17.72
*p* value	0.899	< 0.001	0.497	< 0.001
Partial eta squared (ⴄ^2^)	—	0.200	—	0.183
Diastolic blood				
Control	76.51 ± 11.48	76.86 ± 11.38	75.20 ± 12.13	74.15 ± 12.38
Intervention	74.78 ± 11.35	70.02 ± 10.42	74.34 ± 11.84	67.99 ± 10.66
*p* value	0.517	0.011	0.757	0.026
Partial eta squared (ⴄ^2^)	—	0.087	—	0.068
Systolic blood pressure				
Control	107.54 ± 7.66	104.59 ± 9.82	109.05 ± 9.31	107.16 ± 8.31
Intervention	108.19 ± 8.68	97.84 ± 9.81	110.24 ± 10.46	100.32 ± 10.29
*p* value	0.390	< 0.001	0.704	0.002
Partial eta squared (ⴄ^2^)	—	0.170	—	0.127
Oxygen saturation				
Control	96.16 ± 2.84	96.43 ± 2.14	96.22 ± 2.46	96.43 ± 2.06
Intervention	96.95 ± 2.46	96.73 ± 2.11	96.78 ± 2.02	97.14 ± 2.11
*p* value	0.209	0.986	0.283	0.345
Partial eta squared (ⴄ^2^)	—	0.001	—	0.013

The independent *t*‐test showed that the mean systolic blood pressure was not statistically significantly different between the groups (*p* = 0.390), while ANCOVA showed that after the intervention, the mean systolic blood pressure was significantly lower in the intervention group than in the control group (*p* < 0.001). On the second day, the results of the independent *t*‐test showed that the mean systolic blood pressure between the two groups before the intervention was not statistically significantly different (*p* = 0.704). However, the ANCOVA showed that immediately after the intervention, the mean systolic blood pressure in the intervention group was significantly lower than that in the control group (*p* = 0.002). The effect sizes between groups after the intervention on first day was large (ⴄ2 = 0.170) and on the second day was medium (ⴄ2 = 0.127).

The results of the independent *t*‐test showed that on the first day before the intervention, the mean oxygen saturation of the arterial blood in the two groups did not differ statistically (*p* = 0.209). The ANCOVA also showed that there was no statistically significant difference in mean arterial blood oxygen saturation between the two groups immediately after the intervention (*p* = 0.986). The same trend was observed on the second day, with no statistically significant difference between the two groups before and immediately after the intervention (*p* = 0.283 *and* *p* = 0.345, *r* *e* *s* *p* *e* *c* *t* *i* *v* *e* *l* *y*). The effect sizes between groups after the intervention in first and second days were small (ⴄ2 = 0.001 *and* 0.013, *r* *e* *s* *p* *e* *c* *t* *i* *v* *e* *l* *y*) (Figure 3).

Figure 3Trends in vital signs on the first and second days in the control and intervention groups. (a) Systolic blood pressure. (b) Diastolic blood pressure. (c) Heart rate. (d) Oxygen saturation.

**Figure:**
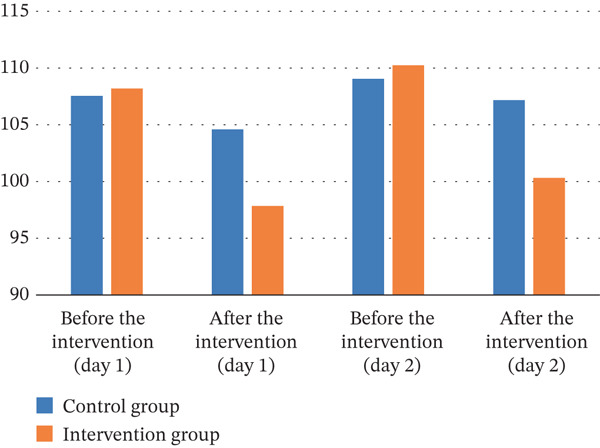
(a)

**Figure:**
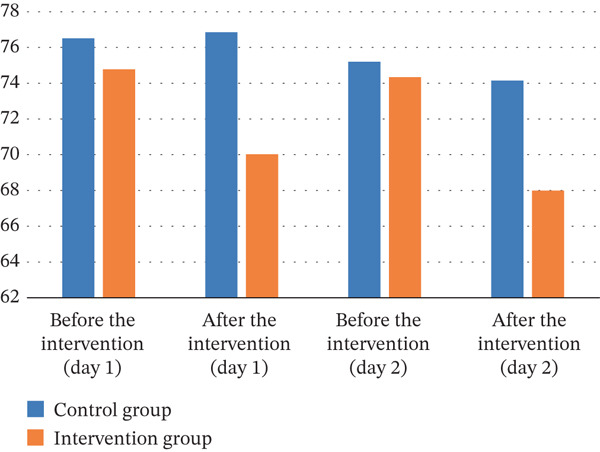
(b)

**Figure:**
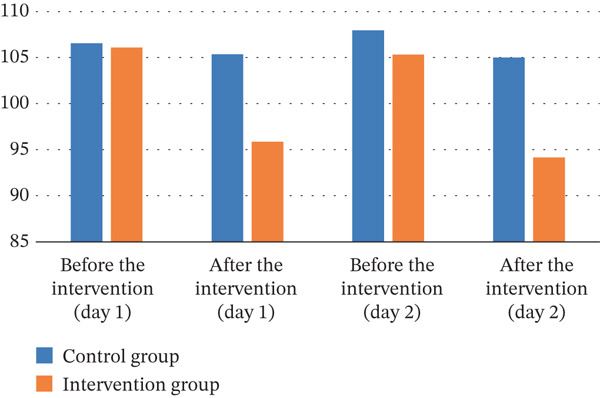
(c)

**Figure:**
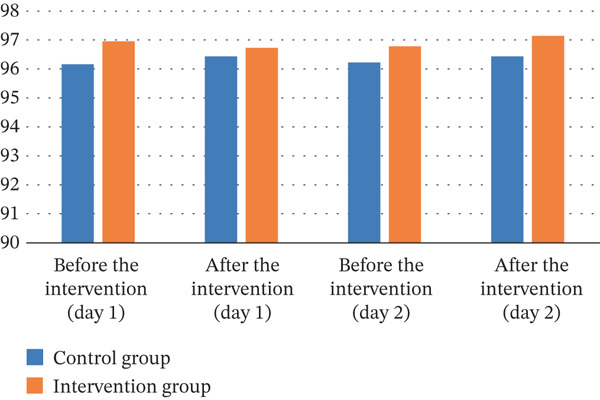
(d)

## 4. Discussion

A key goal of the present study was to examine delirium and physiological indicators in children admitted to the PICU prior to and following foot reflexology massage.

The results revealed that on the first day, all children in both study groups had delirium at two time points before the intervention; instantly after the intervention, the frequency of delirium in the control and intervention groups was not statistically significantly different. No significant difference was observed in the frequency distribution of delirium between pre‐ and postintervention assessments in either the groups. One interpretation of these results is that foot reflexology might be a successful approach in dropping delirium in children, but its effect is not clearly visible immediately after the intervention versus the control group. This conclusion may be clarified by the fact that the influence of foot reflexology on delirium incidence and severity may require more time to fully manifest. The mechanisms of the impact of massage on the nervous and psychological systems include stimulation of certain nerves and increased blood flow, which gradually improve the symptoms of delirium [34]. Some children respond more quickly and clearly to massage, while for others the effects are more noticeable and take longer to appear [35, 36]. Delirium is also a temporary phenomenon that can influence the presentation of symptoms [37]. Consistent with these evidences, the study by Momeni et al. presented that after the intervention, both groups showed a decrease in delirium percentage; nonetheless, this decrease was not statistically significant [38]. In both studies, the mechanisms of the effects of foot massage are discussed similarly. Both note that the effects of massage may take longer to fully manifest, and long‐term improvements may be more apparent than short‐term results. The study by Fu et al. found that massage and aromatherapy did not have a substantial effect on reducing unpleasant behaviors in dementia, which aligns with our findings [39].

**Figure 4:**
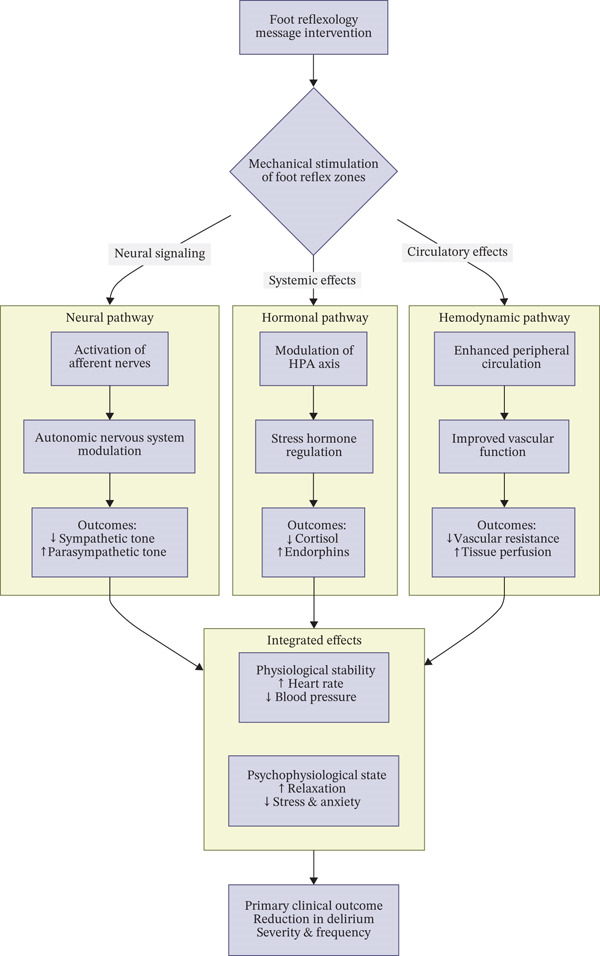
Integrated conceptual model. Proposed mechanisms of foot reflexology on delirium and physiological indicators.

The second‐day analysis revealed a statistically significant disparity in the distribution of delirium incidence between the two groups across both assessed time points. The frequency of delirium reduced significantly following the intervention in contrast to the preintervention period. These findings clearly demonstrate that over time, foot reflexology massage was efficacious in lowering the frequency of delirium in pediatric patients. It appears that foot reflexology massage can help alleviate delirium symptoms by affecting the autonomic nervous system, improving blood flow, and enhancing nerve function. This method can encourage relaxation and alleviate stress through the stimulation of particular reflex points on the feet, which are linked to the central nervous system and various bodily systems. These mechanisms help reduce delirium symptoms and improve the overall condition of children in the PICU [34]. Considering the evidence presented, it is plausible to suggest that the beneficial effects of foot reflexology massage are mediated through a mixture of interconnected mechanisms rather than a single pathway. We propose an integrative model encompassing neural, hormonal, and hemodynamic pathways. From the neural perspective, stimulation of specific reflex zones in the feet may activate afferent nerve fibers and modulate the autonomic nervous system, thereby dropping sympathetic over activity and enhancing parasympathetic tone. From the hormonal perspective, reflexology may influence the hypothalamic–pituitary–adrenal (HPA) axis and other neuroendocrine responses, leading to alterations in stress‐related hormones such as cortisol and endorphins, which promote relaxation and emotional stability [40]. From the hemodynamic perspective, reflexology massage may enhance peripheral circulation, optimize tissue perfusion, and reduce vascular resistance, thereby contributing to improvements in blood pressure and heart rate regulation. Together, these mechanisms form a comprehensive framework that may explain the observed short‐ and long‐term consequences of foot reflexology on both delirium and physiological parameters in critically ill kids (Figure 4).

In conclusion, although the immediate effects of reflexology massage on delirium are not apparent, its long‐term benefits in reducing the frequency of delirium are significant. These evidences suggest the potential of reflexology massage as a nonmedication therapy for handling delirium in hospitalized children [34]. Notably, this study constitutes the initial exploration of the effect of foot reflexology massage on the prevalence of delirium in children. Consistent with these findings, an investigation by Makinian et al. indicated that facial and cranial massage is capable of decreasing the severity of delirium in elderly women hospitalized in the cardiac care department [41]. Putri et al.′s investigation into reflexology‐induced alterations in the mental health of older adults showed that, following the massage, the average scores for depression and anxiety in the elderly decreased, indicating an improvement in their mental health [42].

In contrast to this finding, a study by Fazlollah et al. examined the impact of foot reflexology massage on delirium in adult patients undergoing coronary artery bypass surgery. The results revealed that on the second day after surgery, foot reflexology massage had a significant effect. However, it did not reduce delirium [43]. Important differences in the inclusion and exclusion criteria between our study and study by Fazlollah et al. can clearly explain the mutual effects on the results of both studies. These differences suggest that the more precise and restrictive criteria in the present study may have led to more meaningful results with respect to the influence of massage on delirium.

In our study, results show that immediately afterward the intervention, the mean heart rate in the intervention group was significantly lower than that in the control group. The second‐day data reinforced this pattern, demonstrating a sustained and significant lowering of mean heart rate in the intervention group compared to the control. In other words, foot reflexology significantly reduced heart rate in the intervention group. Consequently, these findings emphasize that foot reflexology massage as a nonmedication therapy method can have significant positive effects on reducing heart rate and improving the physiological condition of children.

In conformity with these results, a study directed by Elsheshtawy et al. revealed that the intervention group exhibited significant improvements in physiological displays (such as heart rate, blood pressure, breathing rate, and temperature). The study recommended that nurses use foot reflexology as a no‐drug approach to improve the physiological status of children undergoing hemodialysis [44]. In addition, evidence from Kotruchin et al. indicates that foot reflexology can significantly reduce heart rate in patients suffering from hypertension [45].

In accordance with these results, a study by Ali Elsabely et al. showed that foot massage significantly improved physiological indicators, for instance, reducing heart rate and blood pressure in the experimental group. Specifically, heart rate decreased significantly in the intervention group [46]. Additionally, the study by Ghaljaei and Jalalodini demonstrated that foot reflexology massage exerted a significant positive effect on key physiological parameters, notably blood pressure and heart rate [47].

The results showed that there was no statistically significant difference in mean diastolic blood pressure between the two groups on the first day before the intervention. Nonetheless, instantly upon initiation of the intervention, the intervention group exhibited a significantly lower mean diastolic blood pressure relative to the control group. Furthermore, this significant difference persisted directly after the intervention′s completion. Consistent with present study, Lin et al. revealed that patients who received Taichung point compression experienced significant reductions in systolic and diastolic blood pressure at 15 and 30 min relative to the control group [48]. Kotruchin et al. also documented significant decreases in systolic and diastolic blood pressure among the intervention group. However, a significant between‐group difference was confined to the reduction in heart rate. The impact of reflexology on lowering blood pressure was less pronounced than in the control group. In conclusion, this study demonstrates that foot reflexology can partially aid in reducing blood pressure in individuals with high blood pressure [45]. However, another study conducted on Korean patients with essential hypertension showed that foot reflexology only decreased systolic blood pressure and did not have any impact on diastolic blood pressure [49]. These findings are at variance with the results reported here. These inconsistencies may be qualified to methodological divergences in reflexology techniques among different trials. Therefore, to advance the field, subsequent experimental studies should systematically compare technique variations and identify the optimal combination of pressure point, strength, and duration for maximizing blood pressure–lowering efficacy. Additionally, foot reflexology massage on the second day was found to effectively reduce diastolic blood pressure in the intervention group. In agreement with these results, a study by Quattrin et al. found that a drop in blood pressure and breathing rate led to a significant improvement in the physiological state of cancer patients. This may help facilitate relaxation and reduce stress in this group of patients [50]. Kuhn et al. believe that reflexology relaxes overactive areas of the body and stimulates underactive areas, thereby helping to balance and relax the body as a whole [17]. Furthermore, Fritz demonstrated that foot manipulation in reflexology stimulates the action of the parasympathetic nervous system. Additionally, the positive effects of massage therapy on improving vital signs may be attributed to its impact on the autonomic nervous system [51].

Our investigation found that sole reflex massage was associated with a substantially greater decline in systolic blood pressure in the intervention group when compared to the control group. Park et al.′s study similarly found that while reflexology lowered systolic blood pressure, diastolic blood pressure remained unchanged [49]. A study by Lestari et al. examined the effect of foot reflexology massage on diastolic blood pressure in patients with high blood pressure. The study showed that after the intervention, the average systolic blood pressure decreased from 160.44 to 140.83 mmHg. This decrease in systolic blood pressure was statistically significant and indicated the positive effect of foot reflexology massage on reducing systolic blood pressure [52]. In explaining this finding, the effective impact of foot reflexology massage on systolic blood pressure may be attributed to its direct effects on the autonomic nervous system and vascular function. Reflexology massage could reduce vascular tension and improve blood flow, ultimately resulting in a decline in systolic blood pressure. Furthermore, this technique may enhance parasympathetic function, which is crucial for regulating blood pressure and promoting relaxation in the body [49]. Research has also found that relaxation and massage techniques can help reduce cortisol secretion and improve hormonal regulation. On the first day, Ann et al. detected no statistically significant difference in either systolic or diastolic blood pressure between the two groups. However, on the second day, significant differences in systolic and diastolic blood pressure were observed. These differences persisted on the third day and remained significant between groups [53].

Finally, our evidences revealed that foot reflex massage had no significant effect on arterial blood oxygen saturation in children. This lack of significant change may be because foot reflexology massage does not significantly impact arterial blood oxygen saturation over a short period. Additionally, slight changes in oxygen saturation were observed in both groups. In the control group, a slight surge was noted, while in the experimental group, a decrease was observed. These changes may have been influenced by other factors such as the initial physiological state or other random effects. Consistent with these results, in their systematic review encompassing three nonclinical trials, Song et al. reported that foot reflexology had a statistically significant positive effect on subjective outcomes like stress, fatigue, and depressive symptoms. However, no statistically significant improvements were noted in objective measures, including blood pressure and heart rate [54]. The results of the Hayes study also showed that a 5‐min foot massage had no effect on patients′ arterial blood oxygen saturation. This result could be attributed to the wide variation in clinical conditions and various diseases within the research community, as well as the absence of a control group [35].

### 4.1. Strengths and Limitations

A key methodological strength was the careful design of a randomized clinical trial and its implementation by a professional multidisciplinary team. However, a limitation was the presence of environmental factors, such as uncontrollable noise in the PICU, which could interfere with the effect of foot reflexology massage. To address this, interventions were implemented, such as asking the treatment team to speak quietly, minimizing noise, and silencing unnecessary alarms. Additionally, in this study, delirium in children was classified as either present or absent without considering its intensity, duration, or the role of hypoactive or hyperactive subtypes. The severity of delirium was assessed only on 2 days. It is recommended that patients be assessed at subsequent intervals to evaluate the stability of the effect.

## 5. Conclusion

Based on this single‐center experience, foot reflexology shows promise as a potential benefit on children′s delirium, showing promise as a nonpharmacological intervention. This finding is clinically significant for nursing care, as reducing delirium without the use of drugs is a significant goal that can decrease medication‐related complications. However, given the constraints of this study, including its single‐center design and short‐term follow‐up, these results should be interpreted as preliminary evidence. Therefore, rather than recommending routine practice, it is more accurate to state that this study provides promising evidence that warrants replication in larger, multicenter trials with longer follow‐up periods to confirm efficacy and establish generalizability. Future research should also examine the impact of the intervention in different age groups. If further robust studies confirm these benefits, foot reflexology could be considered a simple, safe, and low‐cost method for nurses to teach to participants and their families.

## Author Contributions

Z.M.: data curation, investigation, and methodology; M.A‐N.: conceptualized the study, methodology supervisor, developed an analytical framework, validation, and funding acquisition; A.M.A.: data collection supervisor, supervision, validation, and visualization; F.H‐B.: software, formal analysis, writing—review and editing.

## Funding

The study is supported by the deputy of research and technology of Iran University of Medical Sciences, 1403‐1‐3‐3016.

## Disclosure

All authors agree to be accountable for the research presented.

## Ethics Statement

Ethical approval for the study was granted by the institutional ethics committee (Reference Code IR.IUMS.REC.1402.986). The research is also registered on the Iranian Registry of Clinical Trials (IRCT20230202057303N3, accessible at https://irct.behdasht.gov.ir/). Ethical approval was secured, and written informed consent was obtained from each parent or legal guardian after a complete verbal and written explanation of the study′s purpose. All participants were explicitly informed of their voluntary involvement, right to withdraw, and the strict confidentiality protocols governing their data.

## Conflicts of Interest

The authors declare no conflicts of interest.

## Data Availability

The data that support the findings of this study are available from the corresponding author upon reasonable request.
